# FGFR inhibitor, AZD4547, impedes the stemness of mammary epithelial cells in the premalignant tissues of MMTV-ErbB2 transgenic mice

**DOI:** 10.1038/s41598-017-11751-7

**Published:** 2017-09-12

**Authors:** Qingxia Zhao, Amanda B. Parris, Erin W. Howard, Ming Zhao, Zhikun Ma, Zhiying Guo, Ying Xing, Xiaohe Yang

**Affiliations:** 1 Julius L. Chambers Biomedical/Biotechnology Research Institute, Department of Biological and Biomedical Sciences, North Carolina Central University, North Carolina Research Campus, Kannapolis, North Carolina USA; 2 Basic Medical College of Zhengzhou University, Zhengzhou, Henan P.R. China; 3 College of Medicine, Henan University of Sciences and Technology, Luoyang, P.R. China

## Abstract

The fibroblast growth factor receptor (FGFR) family of receptor tyrosine kinases (RTKs) regulates signaling pathways involved in cell proliferation and differentiation. Currently, the anti-tumor properties of FGFR inhibitors are being tested in preclinical and clinical studies. Nevertheless, reports on FGFR inhibitor-mediated breast cancer prevention are sparse. In this study, we investigated the anti-cancer benefits of AZD4547, an FGFR1-3 inhibitor, in ErbB2-overexpressing breast cancer models. AZD4547 (1–5 µM) demonstrated potent anti-proliferative effects, inhibition of stemness, and suppression of FGFR/RTK signaling in ErbB2-overexpressing human breast cancer cells. To study the *in vivo* effects of AZD4547 on mammary development, mammary epithelial cell (MEC) populations, and oncogenic signaling, MMTV-ErbB2 transgenic mice were administered AZD4547 (2–6 mg/kg/day) for 10 weeks during the ‘risk window’ for mammary tumor development. AZD4547 significantly inhibited ductal branching and MEC proliferation *in vivo*, which corroborated the *in vitro* anti-proliferative properties. AZD4547 also depleted CD24/CD49f-sorted MEC populations, as well as the CD61^high^CD49f^high^ tumor-initiating cell-enriched population. Importantly, AZD4547 impaired stem cell-like characteristics in primary MECs and spontaneous tumor cells. Moreover, AZD4547 downregulated RTK, mTOR, and Wnt/β-catenin signaling pathways in premalignant mammary tissues. Collectively, our data provide critical preclinical evidence for AZD4547 as a potential breast cancer preventative and therapeutic agent.

## Introduction

Effective prevention of breast cancer remains a significant challenge due to the heterogeneous nature of tumors that are influenced by numerous genetic and epigenetic factors. Breast cancer subtypes can be categorized based on hormonal receptor (i.e. estrogen and progesterone receptors) and ErbB2/Her2 statuses. Tamoxifen, a selective estrogen receptor modulator (SERM), has shown clinical success by reducing the risk of estrogen receptor-positive (ER^+^) breast cancers by up to 69% as compared to placebo treatment, although approximately 20% of breast cancers are ER-negative (ER−) and do not respond to SERMs^1, 2^. In breast cancers with amplification of the ErbB2 oncogene, which occurs in nearly 30% of breast cancer cases, receptor tyrosine kinase (RTK) inhibitors targeting epidermal growth factor receptors (EGFRs), like trastuzumab and lapatinib, have demonstrated significant clinical benefits as well^3, 4^. Nevertheless, the development of resistance to cancer preventatives and therapeutics, including tamoxifen, trastuzumab, and lapatinib, has proven a significant challenge^5–7^. Therefore, the need to explore novel agents and therapeutic targets in order to improve current preventative and therapeutic outcomes is critical. To this end, fibroblast growth factor receptors (FGFRs) have emerged as promising targets for anti-cancer therapeutics with particular emphasis on refractory breast cancer subtypes and cases that have developed drug resistance^8^.

The FGFR family of RTKs (FGFR1-4) is activated by interactions with various fibroblast growth factors (FGFs) to regulate signaling pathways involved in cell proliferation, survival, and differentiation^9^. In particular, FGFR activation can stimulate cellular responses through downstream PI3K/Akt, MAPK, and Erk signaling, alongside crosstalk with the canonical Wnt signaling pathway^10, 11^. Dysregulation of these signaling pathways can lead to oncogenic conditions that can also facilitate the development and spread of the disease, including epithelial to mesenchymal transition (EMT) and consequential metastasis^10^. As such, mutations of specific FGFRs are associated with various cancers, including bladder cancer (FGFR3 mutation) and sarcoma (FGFR4 mutation)^9, 12–14^. Likewise, FGFR1 upregulation/overexpression is associated with prostate cancer in men and breast cancer in women^9, 15–17^. Physiologically, FGFRs, especially FGFR1 and FGFR2, play a role in mammary development as it has been previously reported that FGFR signaling is necessary for postnatal mammary gland morphogenesis in mouse models^18, 19^. FGFR1 and FGFR2 are both found in the terminal end buds (TEBs) of developing mammary ducts, which are also rich in mammary stem cells (MaSCs) during morphogenesis^20–22^. In this regard, aberrant FGFR expression and signaling can induce mammary morphogenic and MaSC reprogramming with oncogenic consequences. Hence, FGFR-targeting offers a promising anti-cancer strategy that warrants further investigation.

Several studies have explored the anti-cancer potential of FGFR-selective inhibitors in multiple breast cancer models. In particular, an FGFR1 inhibitor, SU5402, reduced cell survival in MDA-MB-134 (overexpresses FGFR1^23^), MCF7, and ZR-75-1 breast cancer cell lines and CAL51 metastatic breast cancer cell line^24^. Similarly, PD173074, an FGFR1/FGFR3 dual inhibitor, was shown by Koziczak *et al*.^25^ to block cell cycle progression in MDA-MB-453, MDA-MB-415, and SUM52 breast cancer cell lines through modulation of Cyclin D1, while Azuma *et al*.^26^ determined that PD173074 induced apoptosis in a lapatinib-resistant breast cancer cell line (UACC812/LR) via an EGFR-independent mechanism^25, 26^. The promising anti-cancer effects of PD173074 have also been translated into a xenograft tumor model of breast cancer in BALB/c mice where 4T1 xenograft tumor growth was remarkably suppressed by this FGFR1/FGFR3 dual inhibitor. NVP-BGJ398 (BGJ398) is an FGFR1/FGFR2/FGFR3 inhibitor that has exhibited anti-cancer effects in breast cancer cell lines, animal models, and Phase I/II clinical trials^27, 28^. Based on the success of PD173074 and BGJ398, more potent inhibitors of FGFR were developed. Of these newly developed experimental drugs, AZD4547, a small molecule reversible inhibitor of FGFR1/FGFR2/FGFR3, exhibited very low cellular IC_50_ values for FGFR1, FGFR2, and FGFR3 (FGFR1: 12 nM, FGFR2: 40 nM, FGFR3: 2 nM)^29^. Indeed, AZD4547 has demonstrated anti-cancer activity in Phase II/III clinical trials focusing on the regression of breast and gastric tumors^30^. Since AZD4547 targets a broader range of FGFRs, the risk of developing drug resistance via alternative signaling through other FGFRs is minimized. Nevertheless, despite the improved potency of AZD4547 as compared to previous FGFR inhibitors, further studies are necessary to interpret the central mechanisms that elicit the anti-cancer effects of AZD4547 and to determine the potential for use in cancer prevention.

As a potential mechanism for the anti-cancer effects of FGFR inhibition, Pond *et al*.^19^ reported that FGFR signaling is necessary for MaSC function^19^. Therefore, the modulation of MaSC function is a beneficial regulatory consequence of FGFR inhibition and is a target of many other anti-cancer therapeutics. MaSCs are unipotent or bipotent with the potential to differentiate into luminal and basal myoepithelial progenitor cells^31, 32^. As mentioned before, MaSCs are essential for mammary gland development; however, oncogenic transformation in MaSCs also can give rise to cancer stem cells (CSCs)/tumor-initiating cells (TICs), which are deemed the sites of cancer initiation in rodents as well as humans^33, 34^. Indeed, TICs are implicated in aggressive mammary tumor development with poor clinical outcomes^35^. Considering the close association between FGFR signaling activity and MaSC function in mammary morphogenesis, AZD4547 is a promising agent for breast cancer prevention due to its potential MaSC-targeted effects. Nevertheless, the underlying anti-cancer mechanisms of AZD4547 require further investigation.

Previous studies from our lab have tested other potential cancer preventatives, including metformin, in the well-established MMTV-ErbB2 transgenic mouse model^36^. Using ErbB2-overexpressing breast cancer cell and animal models, including MMTV-ErbB2 mice, our current study reveals anti-proliferative effects of AZD4547 treatment *in vitro* and *in vivo*. These effects are accompanied by significant depletion of MaSC populations and self-renewal properties. We also demonstrated that AZD4547, administered for 10 weeks during the risk window for mammary tumor development in MMTV-ErbB2 mice, induced architectural and histological changes in the premalignant mammary tissues. Taken together, the morphogenic, MaSC, and signaling regulation associated with AZD4547 treatment provides critical evidence for its potential as a breast cancer preventative or therapeutic agent.

## Results

### AZD4547 inhibits cell proliferation of ErbB2-overexpressing breast cancer cells *in vitro*

**Figure 1:**
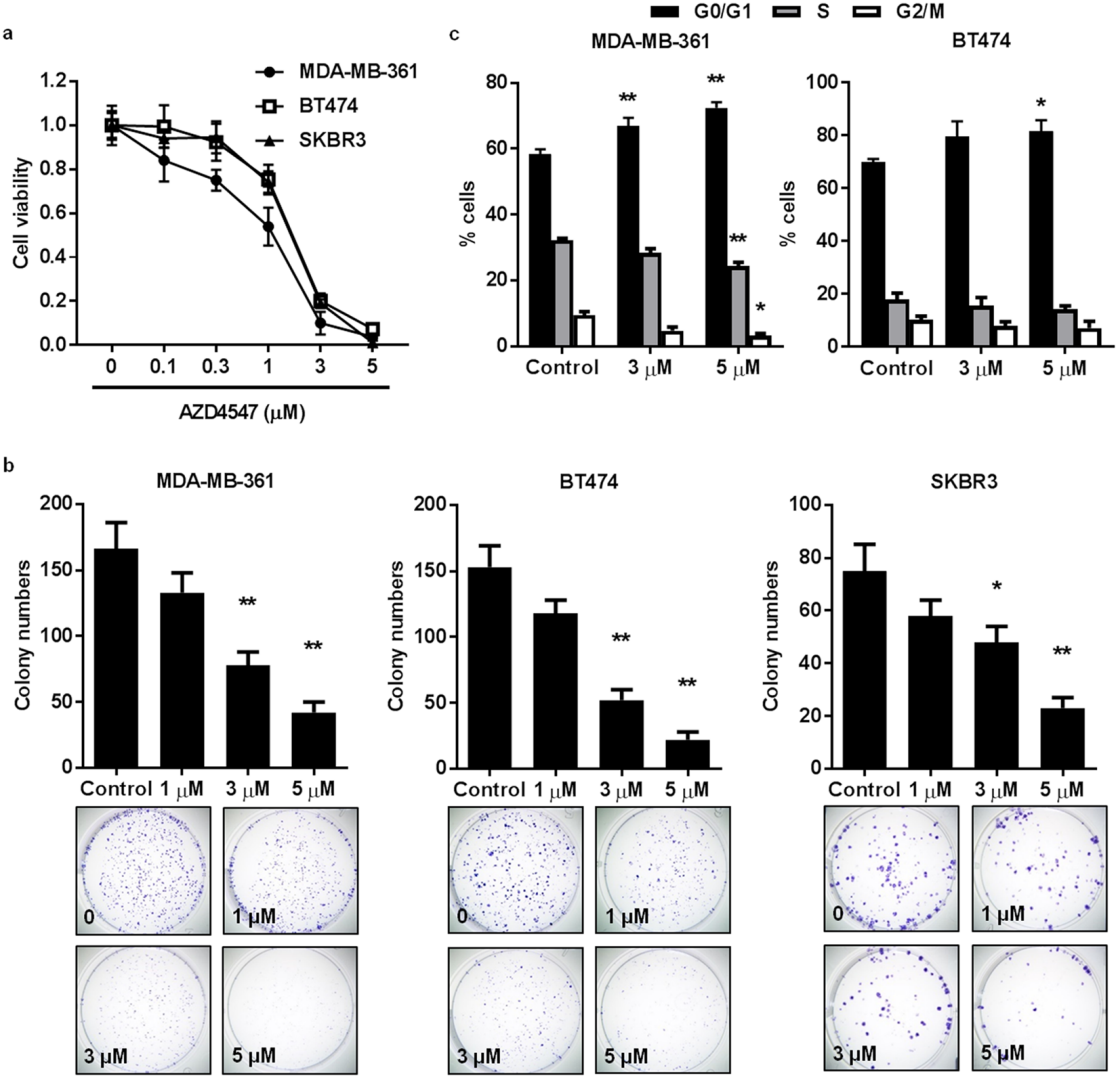
AZD4547 inhibits cell proliferation of ErbB2-overexpressing breast cancer cells *in vitro*. (**a**) MDA-MB-361, BT474, and SKBR3 ErbB2-overexpressing breast cancer cells were treated with AZD4547 (0, 0.1, 0.3, 1, 3, or 5 µM) for 5 days. Then the viable fraction of each cell line was determined with an MTS assay. (**b**) MDA-MB-361, BT474, and SKBR3 cells were treated with AZD4547 (0, 1, 3, or 5 µM) for 12 days before cells were fixed and stained with crystal violet. The number of colonies that formed in each sample is presented in the graphs with representative images in the panels below. (**c**) MDA-MB-361 and BT474 cells were treated with AZD4547 (0, 3, or 5 µM) for 16 hours, then the cells were assessed for the percent of cells in G0/G1, S, and G2/M phase of the cell cycle using FACS analysis. The average percentages of cells in each phase are graphed. Values are presented as the mean ± S.E. (^*^ *P* ≤ 0.05, ^**^ *P* ≤ 0.01 as compared to the corresponding controls).

As uncontrolled cell growth and survival are hallmarks of cancer^37^, we first tested the effects of AZD4547 on the proliferation of ErbB2-overexpressing MDA-MB-361, BT474, and SKBR3 breast cancer cell lines. Data from MTS assays demonstrated that AZD4547 induced dose-dependent inhibition of cell proliferation in the MDA-MB-361 (IC_50_ = 1.18 μM), BT474 (IC_50_ = 1.92 μM), and SKBR3 (IC_50_ = 1.88 μM) cells (Fig. 1a). Moreover, we also determined the effects of BGJ398 and SU5402, two experimental FGFR inhibitors, on cell proliferation as compared to AZD4547. As shown in Supplementary Fig. S1, BGJ398 and SU5402 also induced dose-dependent inhibition of cell proliferation, which was comparable to the effects induced by AZD4547 in Fig. 1a. To note, AZD4547 was marginally more potent, as indicated by lower IC_50_ values, in all three breast cancer cell lines tested. Furthermore, the clonogenic efficiency of these cell lines was suppressed by increasing doses of AZD4547 (Fig. 1b). To further explore the anti-proliferative cellular response to AZD4547, we determined that AZD4547 blocked cell cycle progression with arrest in G0/G1 phase, particularly in the MDA-MB-361 cells (Fig. 1c). Together, these data suggest the anti-cancer potential of AZD4547 through an anti-proliferative mechanism.

### AZD4547 impairs stem cell populations alongside inhibition of RTK signaling *in vitro*

**Figure 2:**
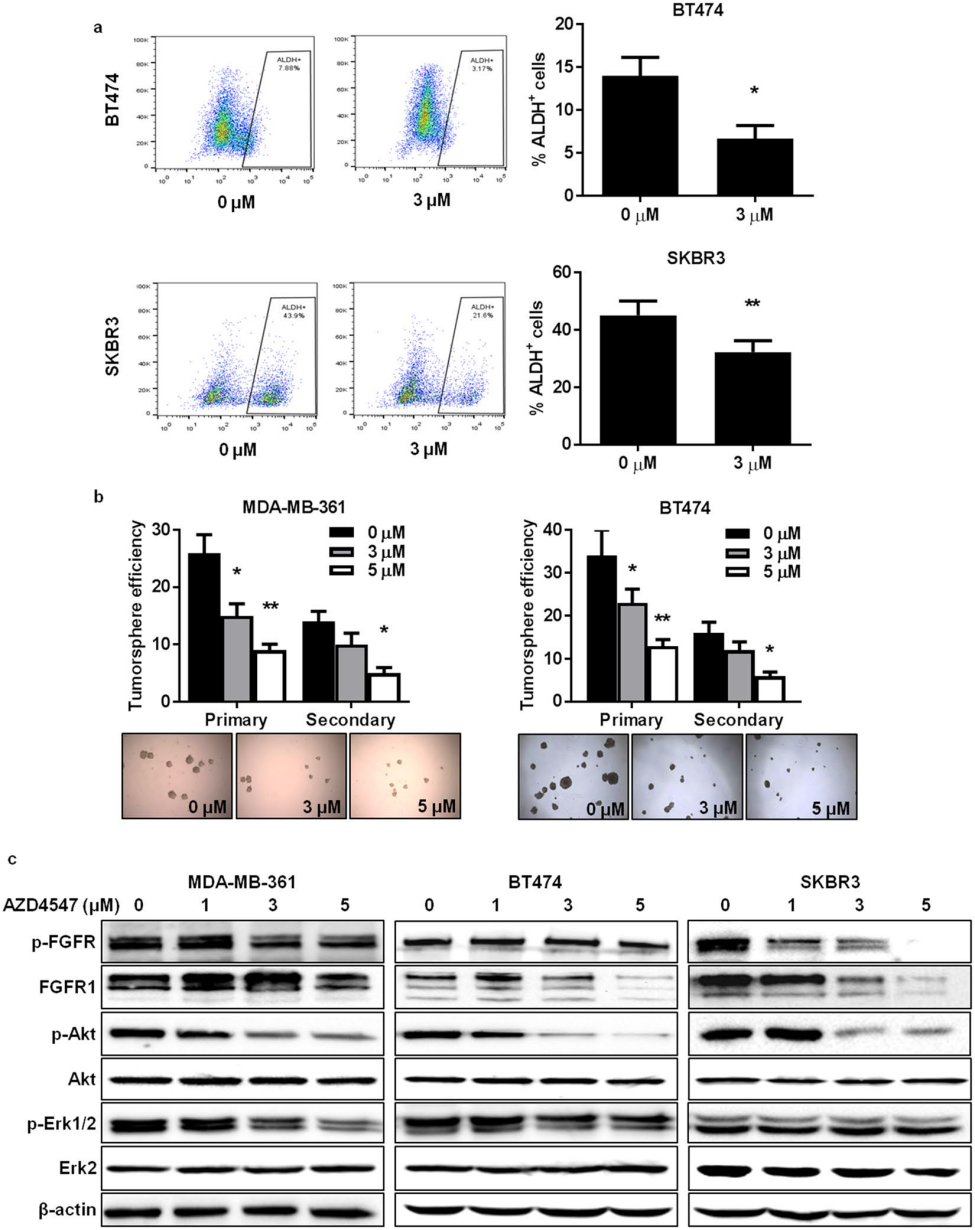
AZD4547 impairs stem cell populations alongside inhibition of RTK signaling *in vitro*. (**a**) BT474 and SKBR3 cells were treated with AZD4547 (0 or 3 µM) for 16 hours. Then, ALDH activity was measured using an ALDEFLUOR assay to determine the percentage of ALDH^+^ cells. Representative flow cytometry plots are presented in the panels to the left. (**b**) MDA-MB-361 and BT474 cells were treated with AZD4547 (0, 3, or 5 µM) for 7 days. Primary tumorspheres were counted and imaged before harvesting. A single cell suspension of primary tumorsphere cells were replated for 7 days to allow secondary sphere formation. The tumorsphere efficiency (number of tumorspheres formed/2000 cells plated) was recorded. Representative images of primary spheres are shown in the panels below the graphs. (**c**) Western blots (cropped) are shown for the indicated proteins in MDA-MB-361, BT474, and SKBR3 cells treated with AZD4547 (0, 1, 3, or 5 µM) for 72 hours. All values are presented as the mean ± S.E. (^*^ *P* ≤ 0.05, ^**^ *P* ≤ 0.01).

The oncogenic transformation of MaSCs into CSCs/TICs is associated with tumorigenesis^21, 32, 35, 38, 39^. Therefore, we explored the effects of AZD4547 on TIC populations and associated self-renewal properties. We found that AZD4547 significantly reduced the percentage of acetaldehyde dehydrogenase-positive (ALDH^+^) cells, which is indicative of the TIC population, in BT474 and SKBR3 breast cancer cells (Fig. 2a). In the primary and secondary tumorsphere formation assay, which measures the self-renewal capacity of the cells, AZD4547 inhibited both primary and secondary tumorsphere formation in a dose-dependent manner in the ErbB2-overexpressing breast cancer cell lines (Fig. 2b). Moreover, we performed Western blot analysis of FGFR and key downstream targets to identify the cellular signaling pathways that may be responsible for these observed effects. As displayed in Fig. 2c, MDA-MB-361, BT474, and SKBR3 cells exhibit FGFR expression and activity, which is consistent with previous reports^40, 41^. Importantly, AZD4547 inhibited FGFR activity with a concurrent and striking downregulation of phospho-Akt and phospho-Erk1/2 expression, which are involved in the regulation of cell growth and survival, in MDA-MB-361, BT474, and SKBR3 cells. In regards to these results, AZD4547 elicits its anti-cancer effects on TIC number and self-renewal function through the suppression of RTK signaling pathways *in vitro*.

### AZD4547 induces histoarchitectural and proliferative downregulation in premalignant mammary glands from MMTV-ErbB2 mice

**Figure 3:**
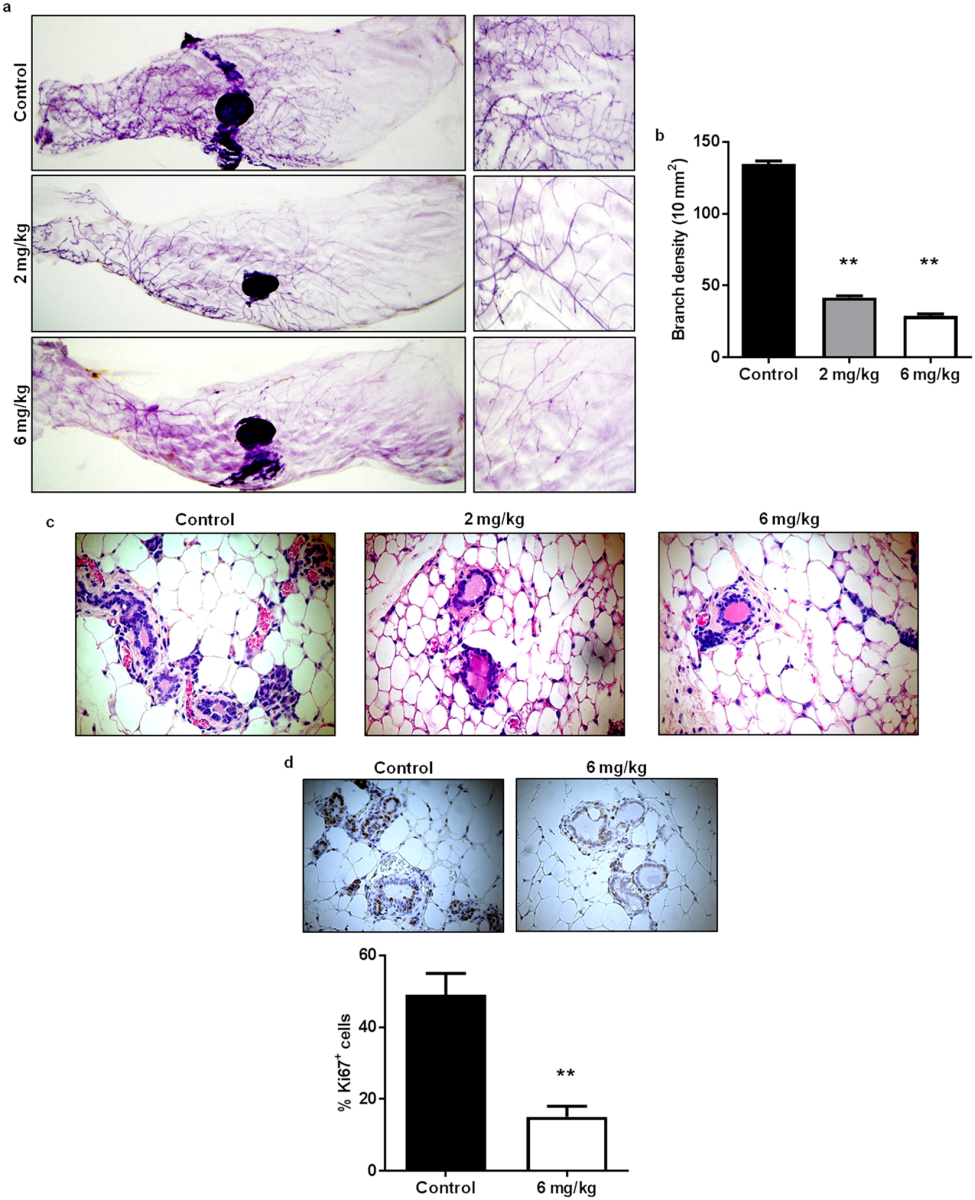
AZD4547 induces histoarchitectural and proliferative downregulation in premalignant mammary glands from MMTV-ErbB2 mice. 8-week-old MMTV-ErbB2 mice were administered AZD4547 (0, 2, or 6 mg/kg/day) for 10 weeks. Then mammary glands were excised for whole mount and histological analyses. Representative images of mammary gland whole mounts are shown in (**a**). Ductal branching complexity (the number of side branches in a 10 mm^2^ field, (**b**) was analyzed from the whole mounts. Four 10 mm^2^ fields were analyzed per sample to determine the branching densities. Mammary glands were also collected after AZD4547 treatments and processed for FFPE. The FFPE tissues were H&E stained (**c**) or processed for IHC staining of Ki67 (**d**). The percentages of Ki67^+^ cells are graphed below the panels of representative Ki67-stained tissues. All values are presented as the mean ± S.E. (^**^ *P* ≤ 0.01).

MaSCs are integral for physiological mammary gland development. Likewise, FGFR1 and FGFR2 are found in the TEBs, which are enriched with MaSCs, of developing mammary glands in adolescent mice and have previously been shown to be necessary for mammary gland maturation^18–20^; however, accelerated mammary morphogenesis and elevated mammographic density are contributors to breast tumorigenesis^42, 43^. To test the effects of FGFR inhibition using AZD4547 in MMTV-ErbB2 mice, we used a prevention model to administer AZD4547 (0, 2, or 6 mg/kg/day) for 10 weeks until the mice reached 18 weeks of age, which is during the premalignant ‘risk window’ of tumor development. To note, the 10-week treatments of AZD4547 did not produce any noticeable toxicities in the mice. Our results demonstrate that AZD4547 induced significant reductions in ductal growth and density as indicated by decreased branching complexity (Fig. 3a,b). Likewise, AZD4547 stimulated histological changes in the mammary gland tissues, as evidenced by decreased thickness of epithelial cell layers forming the duct walls (Fig. 3c). To confirm the changes in the ductal thickness, we performed immunofluorescent (IF) labeling using luminal epithelial markers (K8/18) and basal epithelial markers (K14 and SMA) on mammary tissues from control and AZD4547-treated mice^44^. Indeed, AZD4547 induced an evident decrease in K8/18, K14, and SMA-labeled cells that form the luminal and basal epithelial layers of the mammary ducts (Supplementary Fig. S2). Ki67 staining via immunohistochemistry (IHC) also revealed a significant AZD4547-induced decrease in cell proliferation as compared to the control-treated mice (Fig. 3d). These data translate our *in vitro* findings into our *in vivo* model of ErbB2-overexpressing breast cancer by corroborating the anti-proliferative properties of AZD4547 in MMTV-ErbB2 mice and demonstrate the ability of AZD4547 to alter mammary morphogenesis.

### AZD4547 suppresses stem cell populations and self-renewal in primary mammary epithelial cells (MECs) from MMTV-ErbB2 mice

**Figure 4:**
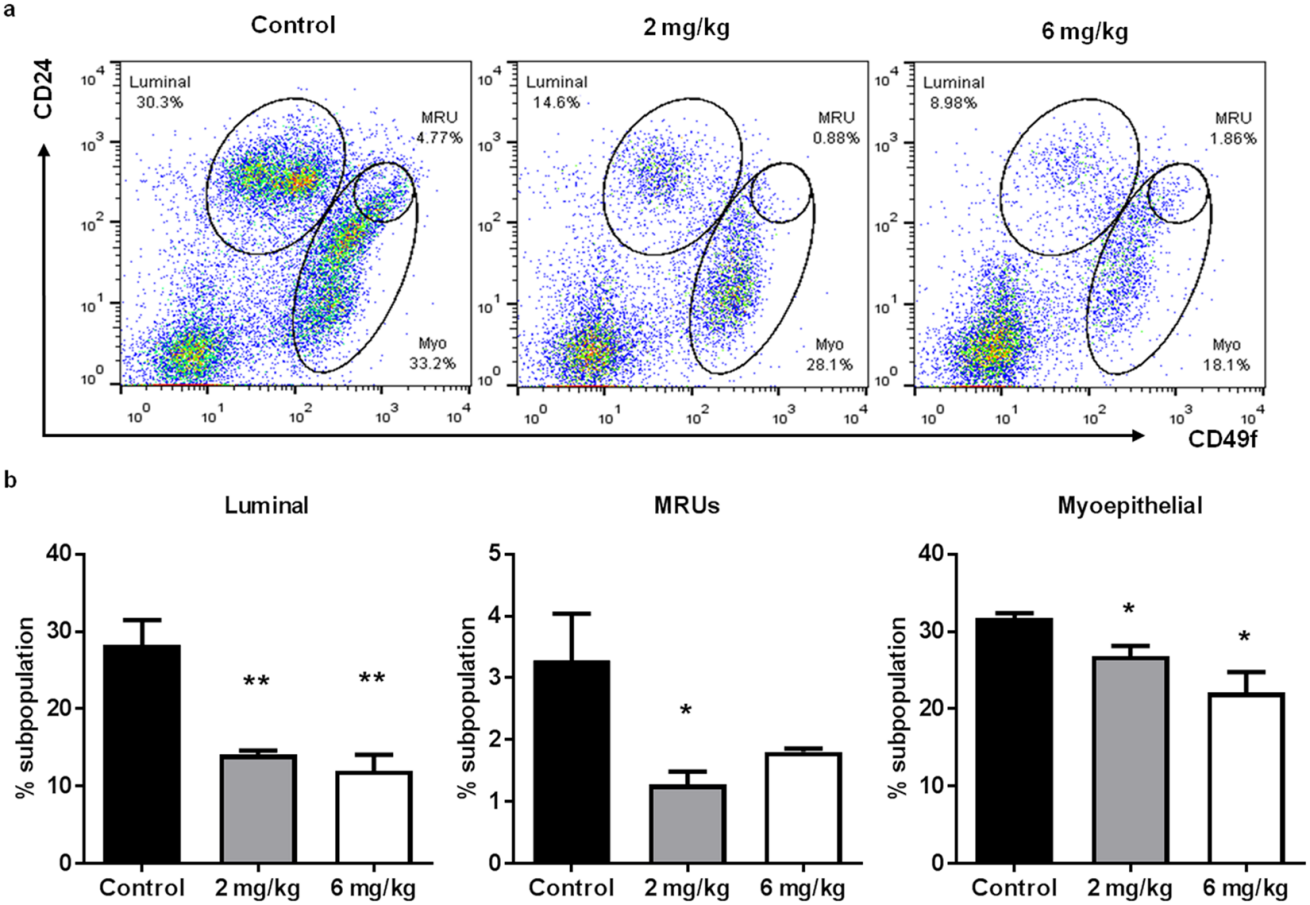
AZD4547 suppresses luminal and MaSC populations in primary MECs from MMTV-ErbB2 mice. Primary MECs were isolated from 18-week-old mice that were treated with AZD4547 (0, 2, or 6 mg/kg/day) for 10 weeks. (**a**) MECs were sorted based on CD24/CD49f expression and the cells formed distinct cell populations of luminal, MRU, and myoepithelial cells. (**b**) The average percentages of cells in the luminal (left panel), MRU (middle panel), and myoepithelial (right panel) subpopulations are graphed. All values are presented as the mean ± S.E. (^*^ *P* ≤ 0.05, ^**^ *P* ≤ 0.01).

Since our *in vitro* data suggest that AZD4547 targets TICs with effects on tumorsphere formation, we determined the *in vivo* effects of AZD4547 on MEC populations. To investigate MEC populations associated with MaSCs, we used CD24/CD49f cell markers to delineate individual cell populations, including luminal, myoepithelial, and putative mammary reconstituting units (MRUs) (Fig. 4a). The luminal cell population, which consists of proliferative progenitor cells, and the myoepithelial/basal cell population are differentiated from MaSCs^31, 32^. Of note, the putative MRU population is enriched with MaSCs. Our flow cytometry analysis revealed that AZD4547 remarkably decreased the percentage of cells belonging to the luminal and basal/myoepithelial cell populations, which is consistent with our data indicating the AZD4547-induced reduction in the thickness of the epithelial cell layers of the mammary ducts. AZD4547 also suppressed the putative MRU population (Fig. 4b). The decreases in the luminal and basal MEC populations further reflect MaSC suppression since luminal and basal progenitor cells are derived from MaSCs. Overall, AZD4547 seems to induce a phenotypic shift from the proliferative luminal, basal, and putative MRU populations to the stromal cell population (CD24^low^CD49f ^low^).

**Figure 5:**
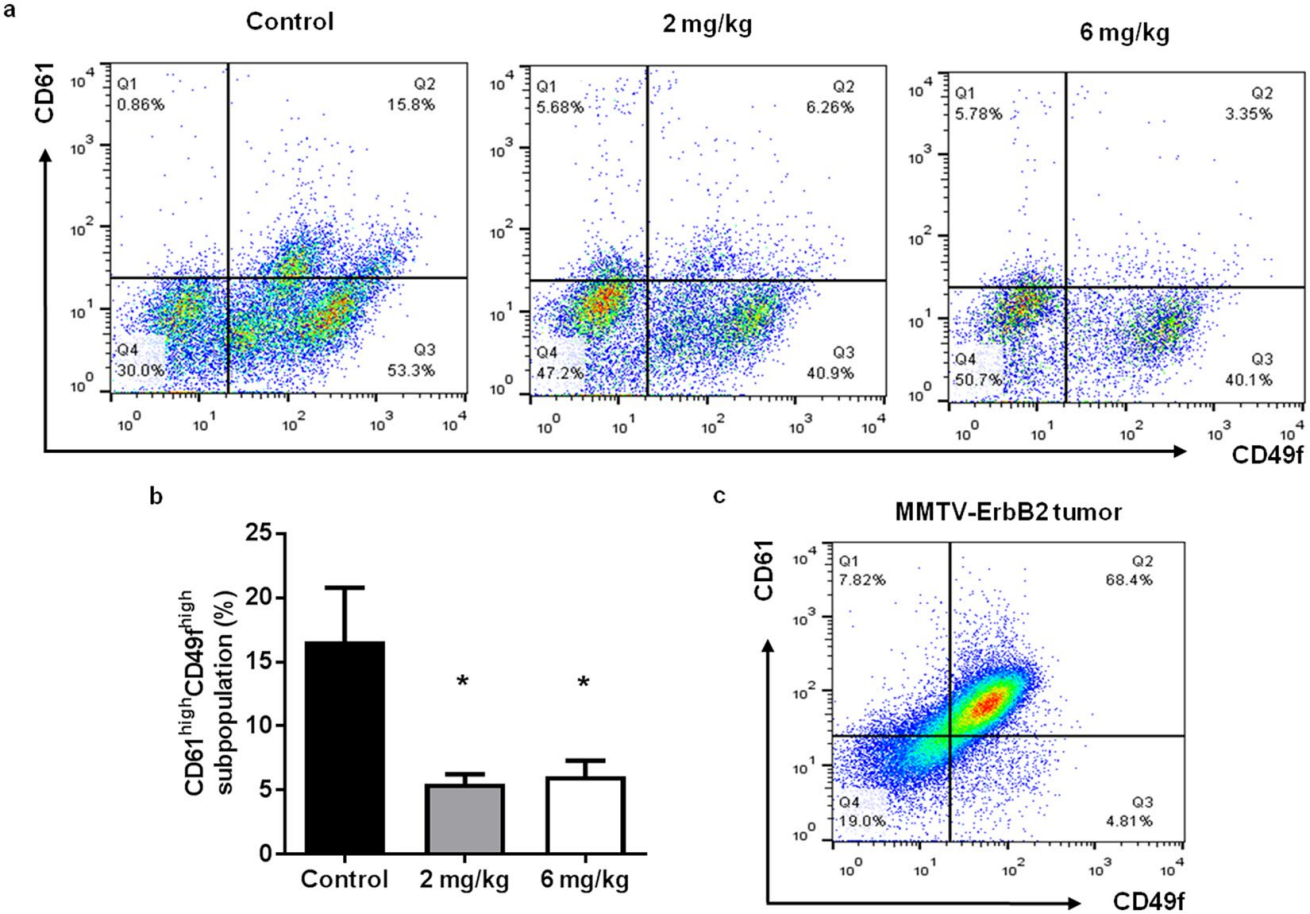
AZD4547 suppresses CSCs/TICs in primary MECs from MMTV-ErbB2 mice. Primary MECs were isolated from 18-week-old mice that were treated with AZD4547 (0, 2, or 6 mg/kg/day) for 10 weeks. MECs were sorted based on CD61/CD49f expression and representative flow cytometry plots are shown in (**a**). The percentages of cells in the CD61^high^CD49f ^high^ population are graphed in (**b**). (**c**) A representative CD61/CD49f flow cytometry plot of untreated primary tumor cells from MMTV-ErbB2 mice with spontaneous mammary tumors is shown. All values are presented as the mean ± S.E. (^*^ *P* ≤ 0.05).

**Figure 6:**
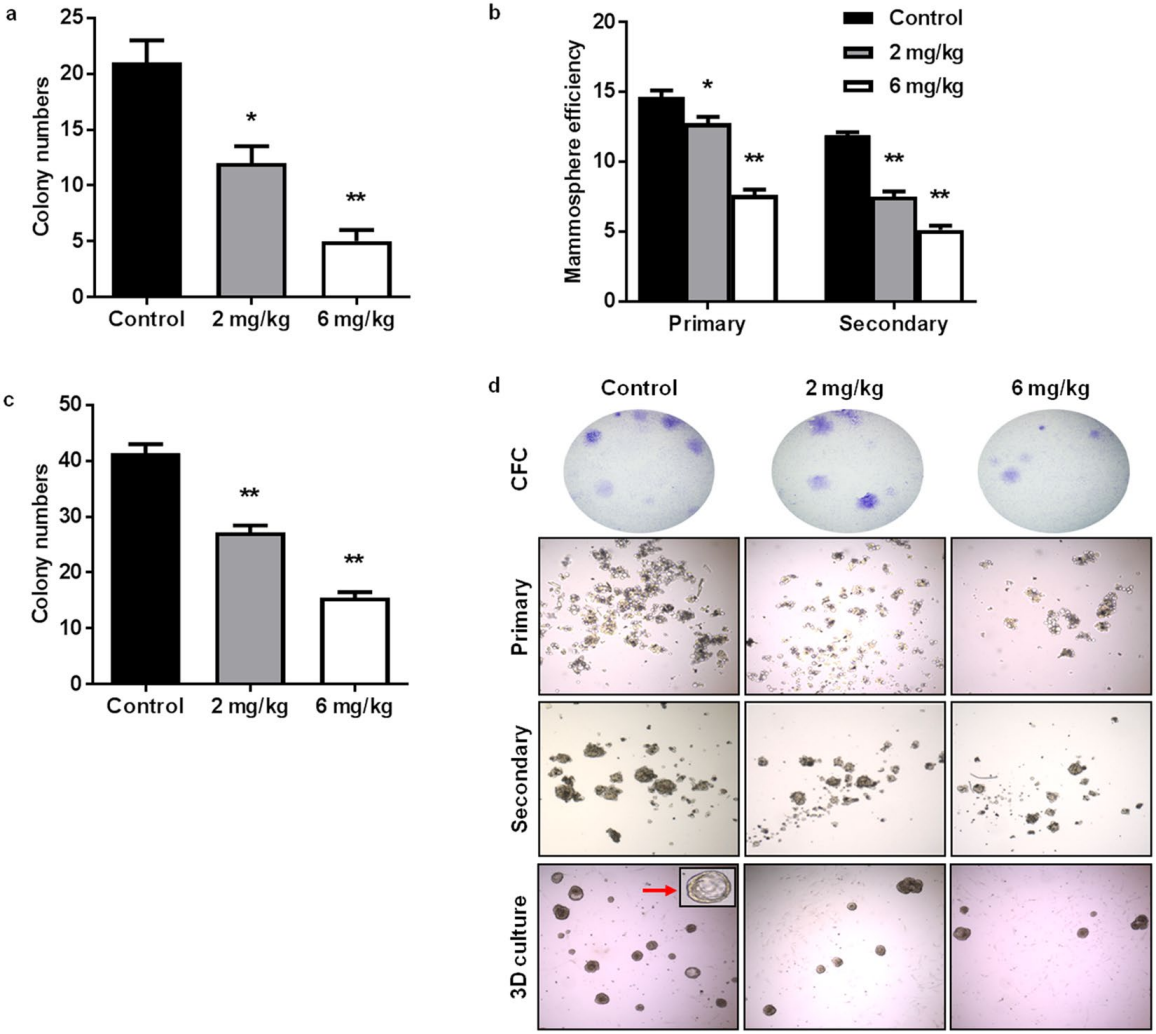
AZD4547 blocks stemness of primary MECs from MMTV-ErbB2 mice. (**a**) Isolated primary MECs were plated for 12 days and stained to determine the colony-forming capacity of the cells. The number of colonies formed from control and AZD4547 (2 or 6 mg/kg/day)-treated mice are displayed. (**b**) Primary MECs were incubated for 7 days to form primary mammospheres. Primary mammospheres were counted and imaged before harvesting. A single cell suspension of primary mammosphere cells were replated for 7 days to allow secondary sphere formation. The mammosphere efficiency (number of mammospheres formed/1000 cells plated) of MECs from control and AZD4547 (2 or 6 mg/kg/day)-treated mice was recorded. (**c**) Isolated MECs were cultured for 7 days in matrigel for a 3D culture assay. The average numbers of colonies formed were recorded. All values are presented as the mean ± S.E. (^*^ *P* ≤ 0.05, ^**^ *P* ≤ 0.01). (**d**) Representative images of colonies and mammospheres from the indicated assays are shown.

MaSCs have the potential to undergo oncogenic mutations and develop into CSCs/TICs, which are highly proliferative cells with self-renewal properties that contribute to the initiation, metastasis, and relapse of breast cancer^32, 35, 39^. Therefore, the regulation of MaSCs is a key anti-cancer target. To further characterize AZD4547-induced MaSC reprogramming, we used alternative stem cell markers (CD61/CD49f) that have been shown to recognize TIC-enriched populations in MMTV-ErbB2 mice^45^. As such, we identified a significant decrease in the CD61^high^CD49f ^high^ population, which represents luminal progenitor cells that have the potential to give rise to TICs^45^, after AZD4547 preventative treatment (Fig. 5a,b; Q2). In Fig. 5c, we confirmed that the tumors from untreated MMTV-ErbB2 mice are comprised of mainly CD61^high^CD49f ^high^ cells. The *in vivo* effects of AZD4547 on stem cell populations were further confirmed using surrogate stem cell functional assays. To this end, AZD4547 suppressed colony formation of primary MECs from treated mice (Fig. 6a), which is indicative of a decreased luminal progenitor population. We also demonstrated that AZD4547 treatment significantly inhibited primary and secondary mammosphere formation, as well as anchorage-independent cell growth in MMTV-ErbB2 mice (Fig. 6b–d). In our model of mammary tumor prevention, our results highlight the AZD4547-mediated inhibition of self-renewing TIC precursors in premalignant mammary glands.

**Figure 7:**
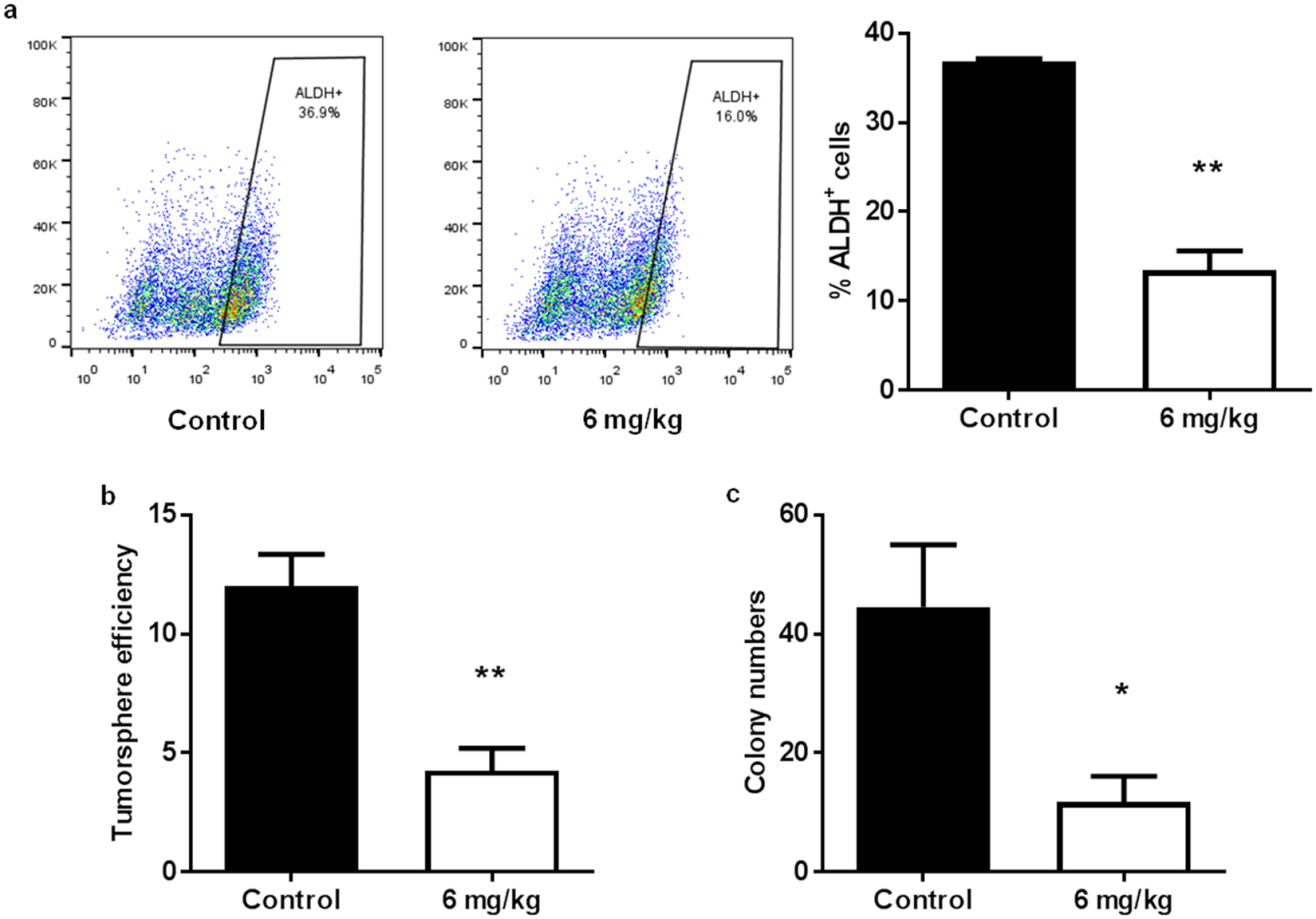
AZD4547 treatment reduces stemness in spontaneous tumors from MMTV-ErbB2 mice. MMTV-ErbB2 mice with spontaneous mammary tumors were treated with AZD4547 (0 or 6 mg/kg/day) for 3 weeks. (**a**) Tumors were excised and primary tumor cells were isolated for an ALDEFLUOR assay of ALDH activity. Representative plots of the ALDH^+^ cell gating are found in the left and middle panels. The percentages of ALDH^+^ cells are graphed in the right panel. (**b**) Primary tumor cells were subjected to a primary tumorsphere assay for 7 days. Then, tumorspheres were imaged and counted. (**c**) Primary tumor cells were also grown in matrigel for a 3D culture assay. The numbers of colonies formed after 7 days are graphed. All values are presented as the mean ± S.E. (^*^ *P* ≤ 0.05, ^**^ *P* ≤ 0.01).

To investigate the therapeutic potential of AZD4547, we treated MMTV-ErbB2 mice with palpable spontaneous tumors with AZD4547 (6 mg/kg/day) for 3 weeks. We found that AZD4547 markedly repressed the TIC population in spontaneous tumors, as measured by the percent of ALDH^+^ tumor cells (Fig. 7a). Likewise, the self-renewal capacity and anchorage-independent growth of these tumor cells were significantly reduced in primary tumorsphere and 3D culture assays, respectively (Fig. 7b,c). Altogether, our stem cell data suggest that AZD4547 selectively targets CSC/TIC populations, thus indicating a mechanism for the anti-cancer potential of AZD4547.

### AZD4547 modulates signal transduction of FGFR/EGFR, mTOR, and Wnt/β-catenin pathways *in vivo*

**Figure 8:**
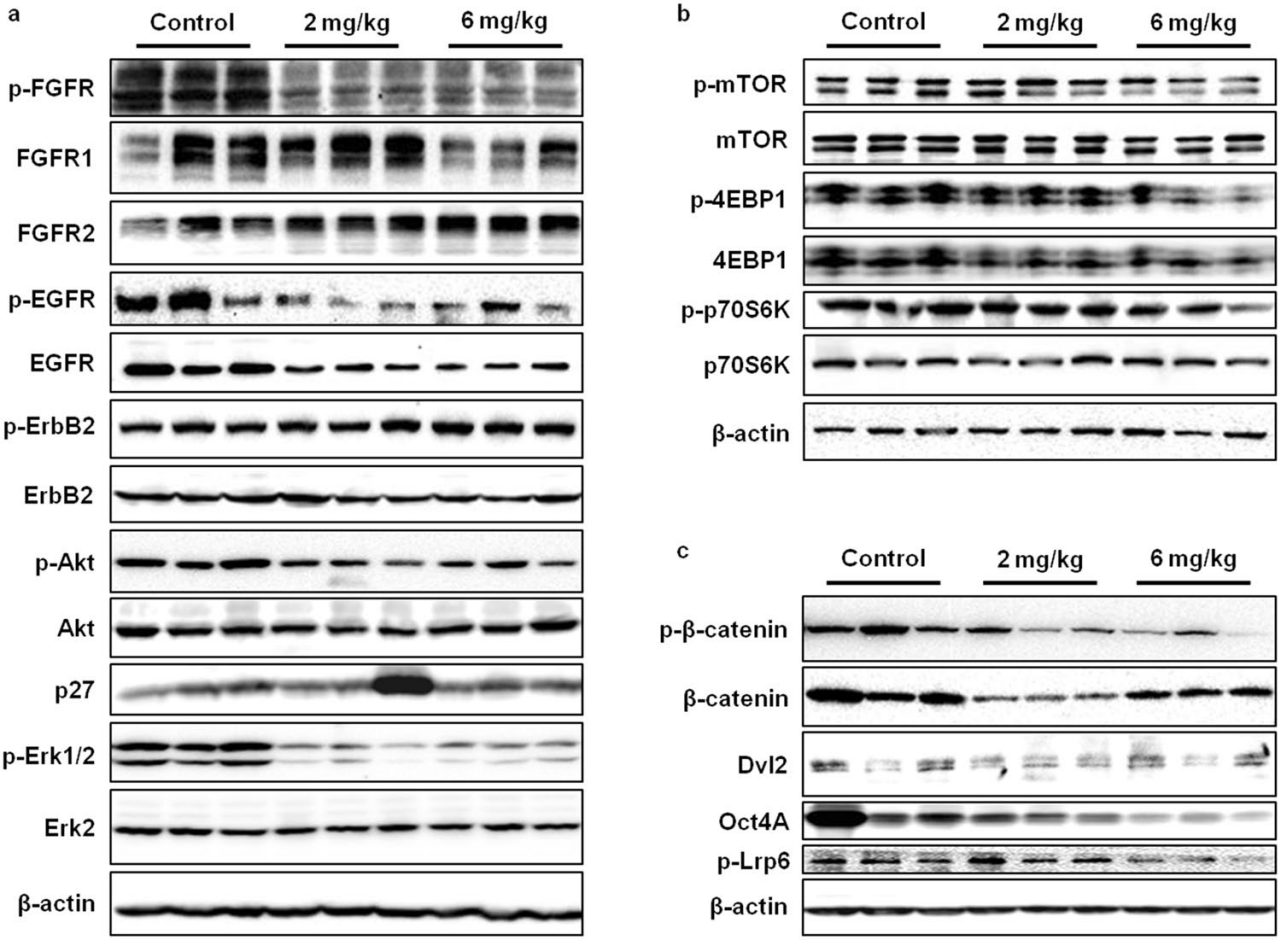
AZD4547 downregulates FGFR/EGFR, mTOR, and Wnt/β-catenin signaling in MMTV-ErbB2 mice. The expression of proteins associated with FGFR/EGFR (**a**), mTOR (**b**), and Wnt/β-catenin (**c**) signaling pathways were measured in mammary gland tissue lysates from control and AZD4547 (2 or 6 mg/kg/day for 10 weeks)-treated mice. Cropped images of Western blots are shown for the indicated proteins.

Identifying the pathways involved in the anti-cancer effects of AZD4547 is essential to understanding its full therapeutic potential. Therefore, we tested the effects of AZD4547 on signaling pathways implicated in cell proliferation and stem cell function. Our data on the effects of AZD4547 on the FGFR signaling pathway *in vitro* were substantiated *in vivo*, in that AZD4547 suppressed FGFR, Akt, and Erk1/2 activation/phosphorylation (Fig. 8a). EGFR protein expression and phosphorylation were also downregulated by AZD4547, suggesting a connection between the EGFR and FGFR families of RTKs in ErbB2-overexpressing breast cancers. As such, this interesting connection will be addressed further in the *Discussion* section. It is also important to note that AZD4547 treatment did not stimulate significant phosphorylation of ErbB2, thus avoiding compensatory activation of downstream signaling, which is associated with acquired resistance. We further found that AZD4547 treatment suppressed the activation/phosphorylation of mTOR and its downstream targets, 4EBP1 and p70S6K, especially at the 6 mg/kg dosage (Fig. 8b). To examine the *in situ* effects of AZD4547 on the mTOR pathways in premalignant mammary tissues using IHC, we determined that 4EBP1 and p70S6K phosphorylation was consistently inhibited by AZD4547 treatment as compared to the control-treated mouse tissues (Supplementary Fig. S3). These decreases in protein activation/phosphorylation involved in the regulation of gene transcription (i.e. mTOR signaling^46^) are concurrent with the downregulation of mRNA expression levels of multiple genes involved in Wnt/stem cell signaling, including *FZD7*, *WNT1*, *CTNNB1*, *APC*, *SFRP1*, *EPCAM*, and *CCND1* (data not shown). In addition to the overall suppression of the RTK-mediated pathways associated with cell growth and survival, the Wnt/β-catenin pathway was inhibited by AZD4547, which provides a mechanism for the stem cell population and functional changes that were induced by AZD4547 in our cell and animal models of ErbB2-overexpressing breast cancer. In particular, a decrease in phospho-β-catenin and Oct4A expression was evident with increasing doses of AZD4547 (Fig. 8c). Taken together, our protein signaling data present several potential pathways that may play a pivotal role in mediating the anti-cancer properties of AZD4547 in mice.

## Discussion

FGFR activity is implicated in various cellular responses associated with mammary tumor initiation/progression, including cell proliferation and survival, as well as mammary gland morphogenesis and MaSC function^9, 20^. Clinically, FGFR amplification occurs in approximately 20% of breast cancers^47, 48^ and FGFR1 protein overexpression has particularly been linked to poor clinical outcomes in patients with the Luminal A breast cancer subtype^49^. As such, the targeted inhibition of FGFRs has emerged as a promising anti-cancer strategy, especially in breast cancers that overexpress FGFRs and/or have developed resistance to EGFR/ErbB2-targeted therapeutics (i.e. lapatinib, trastuzumab, gefitinib, and erlotinib) through compensatory FGFR activation^50–54^. The majority of preclinical studies and active clinical trials testing FGFR inhibitors as a cancer treatment are in combination with other therapeutics^55^, including combinational treatments of lapatinib (EGFR/ErbB2 inhibitor)/PD173074 (FGFR1 inhibitor), gefitinib (EGFR inhibitor)/AZD4547 (FGFR1-3 inhibitor), and AEE788 (pan-EGFR inhibitor)/dovitinib (FGFR1/3 inhibitor)^26, 52, 55, 56^. Despite these extensive studies related to the therapeutic applications of FGFR inhibitors, studies exploring the cancer preventative benefits of FGFR inhibitors are missing.

In this regard, the essential role of FGFRs in mammary development and the potential interactions among RTKs provide the fundamental rationale for investigating FGFR-targeted therapies as chemopreventatives. Since only about 10% of ErbB2-overexpressing breast cancers that have not developed acquired therapeutic resistance also overexpress FGFR, uncovering the preventative application of FGFR inhibition would further broaden the clinical relevance and value of FGFR-targeted agents^57^. As the basis for our current study, the breast cancer preventative capacity of the FGFR1-3 inhibitor AZD4547 remains unclear in individuals who are at-risk for ErbB2-overexpressing breast cancer development due to factors such as: genetics, familial history of breast cancer, personal history of breast cancer, dense mammary tissues, environmental exposures, and lifestyle. To this end, we investigated the anti-cancer mechanisms that are induced by AZD4547 in ErbB2-overexpressing breast cancer models. To note, FGFR protein expression was detected in the cell line (Fig. 2) and animal (Fig. 8) models used in this study.

During our investigation of the *in vitro* anti-cancer mechanisms of AZD4547, we found that AZD4547 suppresses cell proliferation and cell cycle progression in ErbB2-overexpressing breast cancer cell lines (Fig. 1). These anti-proliferative responses to AZD4547 treatment were confirmed by overall FGFR, Akt, and Erk1/2 inactivation (Fig. 2). Consistent with suppressed cell proliferation *in vitro*, AZD4547 reduced ductal branching complexity, suppressed proliferative Ki67^+^ cells, and induced histoarchitectural changes in premalignant mammary glands from MMTV-ErbB2 mice (Fig. 3). AZD4547 also inhibited MaSC and TIC populations alongside impaired self-renewal in ErbB2-overexpressing breast cancer cell lines (Fig. 2), premalignant mammary tissues from MMTV-ErbB2 mice (Figs 4, 5 and 6), and spontaneous tumors (Fig. 7). Our data indicate that AZD4547-stimulated inhibition of the FGFR, EGFR, mTOR, and Wnt/β-catenin pathways (Fig. 8) is associated with *in vivo* cellular responses. AZD4547-induced regulation of Wnt/β-catenin signaling is particularly important since breast cancers that overexpress both FGFR1 and Wnt are correlated with high patient mortality^28^. Therefore, understanding these mechanisms is crucial for the development and optimization of cancer preventative and therapeutic strategies, and has the potential to identify prognostic biomarkers of clinical responsiveness.

Notably, one such indicator of clinical outcomes is the presence of CSCs/TICs, which contribute to tumor heterogeneity, aggressiveness, and recurrence^35, 58, 59^. In our study, we found that AZD4547 selectively inhibited TIC (ALDH^+^ and CD61^high^CD49f ^high^)- and MaSC (putative MRU; CD24^high^CD49f ^high^)-enriched populations both *in vitro* (Fig. 2) and *in vivo* (Figs 4, 5 and 6). The observed reduction in the MaSC and TIC compartments corroborates the *in vivo* downregulation of the Wnt/β-catenin pathway (Fig. 8), which is regarded as a critical regulatory pathway of MaSCs^32, 35, 39, 60^. Our data further suggest that AZD4547 induces MaSC reprogramming, as indicated by reduced luminal, putative MRU, and myoepithelial cell populations, and an apparent increase in the stromal cell (CD24^low^CD49f ^low^) population (Fig. 4). Clonogenicity and sphere formation were also inhibited by AZD4547 treatment *in vitro* (Fig. 1) and *in vivo* (Fig. 6). In the premalignant mammary tissues, these dose-dependent effects on colony-forming cell (CFC) and mammosphere formation are indicative of impaired luminal progenitors (precursors of TICs) and MaSC self-renewal, respectively. Furthermore, we investigated the therapeutic effects of AZD4547 on established spontaneous mammary tumors, which are enriched with TICs (Fig. 5), from MMTV-ErbB2 mice. We found that after 3 weeks of AZD4547 (6 mg/kg/day), ALDH^+^ TIC populations were reduced alongside a significant blockade of tumorsphere formation and anchorage-independent cell growth (Fig. 7). This is a critical finding that further substantiates the selective targeting of TIC precursors and TICs in both premalignant and malignant mammary tissues, respectively, as an anti-cancer mechanism of AZD4547.

Collectively, our results indicate that FGFR1-3 inhibition with AZD4547 is a promising anti-cancer strategy that selectively targets MaSC and CSC/TIC populations. AZD4547 also produced significant cancer preventative and therapeutic effects in our cell and animal models of ErbB2-overexpressing breast cancer. Accordingly, our data demonstrate the effects of AZD4547 on mammary morphogenesis and MaSCs in the premalignant mammary glands of MMTV-ErbB2 mice. Moreover, AZD4547 was administered to the mice for 10 weeks during the ‘risk window’ for mammary tumor development and was well-tolerated, which further supports the concept that AZD4547 and other FGFR inhibitors have potential cancer preventative applications for breast cancer subtypes that are not limited to FGFR overexpression. Screening for FGFR expression in at-risk patients may predict the effects of AZD4547 and other FGFR-targeted therapies on mammary morphogenesis and MaSC reprogramming. Future studies focusing on the effects of AZD4547 on tumor latency in MMTV-ErbB2 mice and other breast cancer models, and relative cellular toxicity in normal mice will be performed to further establish FGFR inhibitors for mammary tumor prevention. Ultimately, additional clinical trials are necessary to assess the translational value of AZD4547 as a cancer preventative or therapeutic; nevertheless, in the context of our study and other reports, FGFR-targeting by AZD4547 has promising clinical applications.

## Materials and Methods

### Antibodies and reagents

AZD4547 was purchased from LC Laboratories (Woburn, MA). FGFR1, FGFR2, p-FGFR, EGFR, p-EGFR, ErbB2, p-ErbB2, Akt, p-Akt, p-Erk1/2, mTOR, p-mTOR, 4EBP1, p-4EBP1, p70S6K, p-p70S6K, β-catenin, p-β-catenin, Dvl2, Oct4A, and p-Lrp6 primary antibodies were purchased from Cell Signaling (Danvers, MA). Erk2, p27, and β-actin primary antibodies, BGJ398, and SU5402 were purchased from Santa Cruz Biotechnology (Santa Cruz, CA). Primary antibodies used for IF staining, including Cytokeratin 14 (K14), Cytokeratin 8/18 (K8/18), and α-smooth muscle actin (SMA) were purchased from Leica Biosystems (Buffalo Grove, IL), Developmental Studies Hybridoma Bank (DSHB; University of Iowa, Iowa City, IA), and Sigma (St. Louis, MO), respectively.

### Cell culture

Breast cancer cell lines, MDA-MB-361, BT474, and SKBR3 cells, were purchased from the American Type Culture Collection (ATCC; Manassas, VA). All cells were maintained in DMEM/F-12 culture medium supplemented with 10% FBS, 100 μg/mL penicillin, and 100 μg/mL streptomycin at 37 °C in an incubator with a humidified 5% CO_2_ atmosphere.

### Animals and treatment

All animal procedures in this study were approved by the Institutional Animal Care and Use Committee (IACUC) of the North Carolina Research Campus (NCRC) and were performed according to relevant guidelines and regulations. Female FVB/N-Tg/MMTV-ErbB2 (MMTV-ErbB2) transgenic mice were purchased from Jackson Laboratories (Bar Harbor, ME) and were fed an estrogen-free AIN-93G diet (Bio-Serv; Flemington, NJ). For the prevention model, AZD4547 (2 or 6 mg/kg/day) was administered via intraperitoneal (i.p.) injection to MMTV-ErbB2 mice (n = 5 mice per group) beginning at 8 weeks of age. The AZD4547 treatments were given daily until the mice reached 18 weeks of age. At 18 weeks of age, the mice were euthanized and the mammary gland tissues were collected for whole mounting, histological analysis, Western blotting, and assays for isolated primary MECs.

For the spontaneous tumor treatment model, MMTV-ErbB2 mice (n = 4 mice per group) with spontaneous mammary tumors were administered vehicle or AZD4547 (6 mg/kg/day) via i.p. injection for 3 weeks. Then, the mice were euthanized and the tumors were excised and homogenized for further analysis.

### Cell viability assay

Cells were seeded (1 × 10^3^ cells/well) in 96-well plates for 24 hours. The next day, indicated concentrations of FGFR inhibitors were added to fresh medium and the cells were incubated for 5 days. After 5 days, cell viability was assessed using a CellTiter 96 AQueous Non-Radioactive Cell Proliferation kit (Promega; Madison, WI) according to manufacturer’s instructions. Briefly, MTS reagent was added to the cells for 2 hours, then absorbance at 490 nm was measured using a SynergyMx microplate reader (BioTek; Winooski, VT). The viable cells are expressed as the cell viability fraction of FGFR inhibitor-treated cells as compared to the untreated control cells.

### Clonogenic and CFC assays

Breast cancer cell lines were plated (1 × 10^3^ cells/well) in 6-well plates. The cultured cell lines were treated with AZD4547 after 24 hours. The isolated MECs were plated (4 × 10^3^ cells/plate) in 60 mm plates. The cultured and primary cells were incubated for 12 days. Then, the cultured cells were stained with 0.5% crystal violet (1:1 methanol in H_2_O), while the primary MECs were fixed in 1:1 acetone in methanol and stained with Wright’s Giemsa. Colonies with ≥50 cells were counted and documented for each sample, and images of the stained colonies were captured with the Nikon SMZ 745 T microscope and Nikon Elements Imaging System Software.

### Cell cycle analysis

Cells were seeded (1 × 10^4^ cells/plate) in 60 mm plates for 24 hours before AZD4547 treatments. After incubation for 16 hours, cells were harvested and fixed in 70% ethanol overnight at −20 °C. Next, the cells were washed in PBS and incubated for 30 minutes at 37 °C in a solution with RNase A (0.5 mg/mL) and propidium iodide (PI; 50 μg/mL). The percent of cells in each phase of the cell cycle was then quantified using Guava easyCyte 8 flow cytometer (Millipore; Billerica, MA) with ModFit software.

### ALDEFLUOR assay

For assessing ALDH activity in cultured cell lines and tumors from mice treated with AZD4547 (6 mg/kg/day for 3 weeks), an ALDEFLUOR kit (Stemcell Technologies; Cambridge, MA) was used. The cultured cells were treated with AZD4547 by 24 hours after seeding. Then, the ALDEFLUOR substrate was added to the cells for 30 minutes at 37 °C. The ALDEFLUOR-positive cell population was determined using an ALDH1 inhibitor, diethylaminobenzaldehyde (DEAB), as a negative control for flow cytometry analyses. With the proper gating for ALDEFLUOR-positive cells defined, the experimental samples were analyzed using Guava EasyCyte Flow Cytometer (EMD Millipore) and FlowJo analysis software and the percentage of ALDH^+^ cells was calculated.

### Tumorsphere/mammosphere and 3D culture assays

Cultured cells and primary MECs were seeded (2 × 10^3^ cells/well and 1 × 10^3^ cells/well, respectively) in ultra-low attachment 6-well plates (Corning) as previously described^61^. After 24 hours, the cultured cells were treated with indicated doses of AZD4547. Then the cells were incubated for 7 days for primary sphere formation. The primary spheres were counted and imaged after 7 days of growth and collected to form a single cell suspension. This single cell suspension was then replated in new ultra-low attachment 6-well plates and incubated for another 7 days. The secondary spheres were counted and imaged. Spheres ranging from 40 to 120 µm in diameter were included in the primary and secondary sphere counts and were used to determine the sphere efficiency (i.e. the number of spheres formed/number of cells plated) for each sample.

For the 3D culture assay, primary MECs and tumor cells from control and AZD4547-treated MMTV-ErbB2 mice were seeded (15000 cells/well) in matrigel in 48-well plates for 7 days. After 7 days, the spheroid cultures were counted and imaged to determine the number of colonies formed.

### Western blot analysis

Protein concentrations of lysates from cultured cell lines and mammary gland tissues were quantified using a BCA Protein Assay kit (Thermo Scientific; Rockford, IL). Equal amounts of protein (50 µg) were separated using 10% or 12% SDS-PAGE and were then transferred to nitrocellulose membranes. After blocking in 5% milk for 2 hours at room temperature, the membranes were incubated in diluted primary antibodies overnight at 4 °C. The next day, the membranes were washed in TBST buffer before incubation for 1 hour at room temperature in appropriate horseradish peroxidase (HRP) secondary antibodies. After final washes in TBST buffer, SuperSignal West Pico ECL solution (Thermo Scientific) was added to the membranes to enhance the chemiluminescent signal. Proteins bands were detected and imaged using the FluorChemE imaging system.

### Whole mount analysis

Whole mounts were prepared from harvested mammary glands of 18-week-old MMTV-ErbB2 mice as previously described^61^. The tissues were spread on glass slides and fixed in Carnoy’s solution overnight at room temperature, followed by rehydration in serial concentrations of ethanol. Afterwards, the tissues were staining with carmine alum overnight. The tissues were then dehydrated, cleared, and mounted on the slides using Permount (Thermo Scientific). Whole mounts were analyzed for histoarchitectural changes induced by AZD4547 with a Nikon Eclipse 80i microscope and the Nikon Elements Imaging System (Nikon Instruments, Inc.).

### Histology, IHC, and IF

Mammary gland tissues from 18-week-old MMTV-ErbB2 mice were formalin-fixed and paraffin-embedded (FFPE) on glass slides, deparaffinized with xylene, and rehydrated with ethanol according to standard procedures. For histological analysis, the FFPE tissues were hematoxylin and eosin (H&E) stained by staining with hematoxylin for 5 minutes and then incubation in consecutive washes of water, acid alcohol (1% HCl in ethanol), and 0.2% ammonia. The tissues were then stained with eosin Y for 1 minute prior to final dehydration, clearing, and mounting with ethanol, xylene, and Permount, respectively.

For IHC analysis of FFPE mammary gland tissues, slides were deparaffinized and rehydrated as mentioned above. Then, the slides were boiling in citrate buffer (pH 6.0) for 30 minutes at 100 °C for antigen retrieval and endogenous peroxidase activity was blocked with 3% H_2_O_2_ in methanol for 10 minutes at room temperature. Horse serum (10%) was used for blocking of nonspecific binding before the slides were incubated overnight at 4 °C in the indicated primary antibodies. The next day, slides were incubated in appropriate secondary antibodies for 1 hour, followed by exposure to the ABC reagent (Vector Laboratories; Burlingame, CA) and diaminobenzidine (DAB). Slides were counterstained with hematoxylin and mounted with Permount for observation. Images of stained sections were captured using the Nikon Eclipse 80i microscope and Nikon Elements Imaging System Software.

For IF analysis of FFPE mammary gland tissues, slides were deparaffinized and rehydrated as describe above. Following antigen retrieval, nonspecific binding was blocked by incubating the slides in 10% horse serum for 30 minutes at room temperature. The slides were then incubated in the indicated primary antibodies overnight at 4 °C, followed by washing in PBST and incubation in either Alexa Fluor 488 goat anti-rat or Alexa Fluor 546 goat anti-mouse secondary antibodies for 1 hour at room temperature in the dark. Then, the slides were washed in PBST, nucleic were stained with DAPI, and the slides were mounted. Slides were observed and imaged using the Nikon Eclipse 80i fluorescence microscope.

### Primary MEC isolation and flow cytometry analysis

Mammary glands were excised from 18-week-old MMTV-ErbB2 mice and primary MECs were isolated as previous described by our lab^36^. Briefly, homogenized tissues were digested in collagenase (Roche) and hyaluronidase (Sigma), followed by further digestion using trypsin-EDTA (Sigma) and dispase (Stemcell Technologies)/DNase I (Sigma). The single cell suspension was then filtered using 40 µm mesh strainers. For flow cytometry analyses, the isolated primary MECs were stained with lineage markers and either CD61/CD49f or CD24/CD49f antibodies according to the procedure defined by Shelton *et al*.^62^.

### Statistical analysis

To determine statistical differences between two groups, a Student’s t-test was performed using GraphPad Prism software (La Jolla, CA). A *P*-value of 0.05 (*P* ≤ 0.05) was chosen for significance.

## Electronic supplementary material

Supplementary Information
