# Supplementary material for: FGFR inhibitor, AZD4547, impedes the stemness of mammary epithelial cells in the premalignant tissues of MMTV-ErbB2 transgenic mice
Source: Sci Rep. 2017 Sep 12;7:11306. doi: 10.1038/s41598-017-11751-7 (PMC5595825; doi:10.1038/s41598-017-11751-7)

FGFR inhibitor, AZD4547, impedes the stemness of mammary epithelial cells in the premalignant tissues of MMTV-ErbB2 transgenic mice

Qingxia Zhao[1,2‡], Amanda B. Parris[1‡], Erin W. Howard[1], Ming Zhao[1], Zhikun Ma[1,3], Zhiying Guo[1], Ying Xing[2], Xiaohe Yang[1,3]*

[1]Julius L. Chambers Biomedical/Biotechnology Research Institute, Department of Biological and Biomedical Sciences, North Carolina Central University, North Carolina Research Campus, Kannapolis, North Carolina

[2]Basic Medical College of Zhengzhou University, Zhengzhou, Henan, P.R. China

[3]College of Medicine, Henan University of Sciences and Technology, Luoyang, P.R. China

*xyang@nccu.edu

‡Authors contributed equally to this work

Supplementary Figure Legends

Supplementary Figure S1: Inhibition of cell proliferation induced by the FGFR inhibitors, BGJ398 and SU5402, in ErbB2-overexpressing breast cancer cells *in vitro*. MDA-MB-361, BT474, and SKBR3 ErbB2-overexpressing breast cancer cells were treated with BGJ398 (0, 0.1, 0.3, 1, 3, or 10 µM) or SU5402 (0, 0.1, 0.3, 1, 3, or 10 µM) for 5 days. Then the viable fraction of each cell line was determined with an MTS assay. The IC_{50} values of BGJ309 and SU5402 are indicated for each cell line.

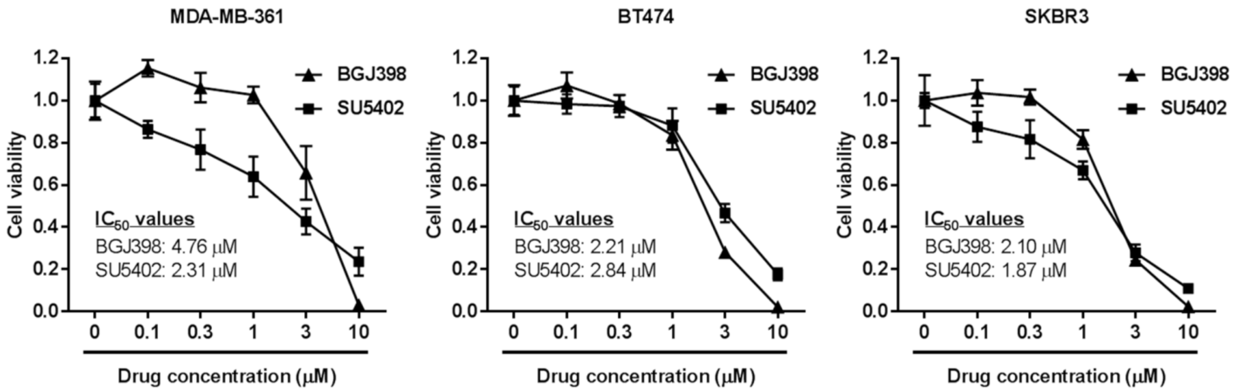

Supplementary Figure S2: AZD4547 reduces the luminal and basal epithelial cell layer thickness of the mammary ducts. The ductal epithelial thickness was compared in control and AZD4547 (2 or 6 mg/kg/day)-treated MMTV-ErbB2 mice. Using IF, the tissues were stained with K8/K18 (**a**) luminal epithelial markers and K14 (**b**) and SMA (**c**) basal epithelial markers (200× total magnification).

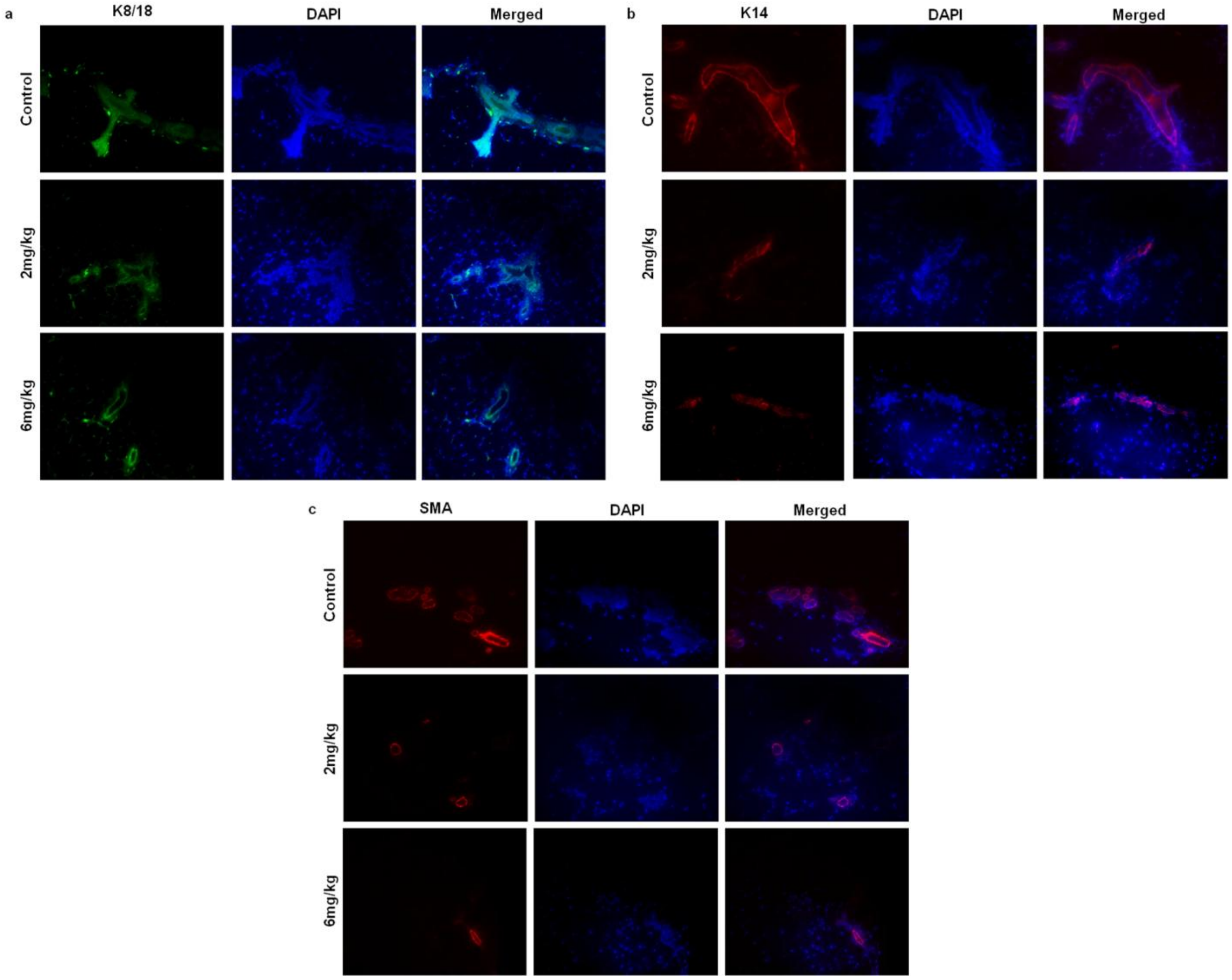

Supplementary Figure S3: AZD4547 suppresses 4EBP1 and p70S6K activation/phosphorylation. The expression and phosphorylation of indicated proteins were analyzed from mammary tissues of control and AZD4547 (6 mg/kg/day for 10 weeks)-treated MMTV-ErbB2 mice using IHC. Brown staining indicates the presence of the specific protein.

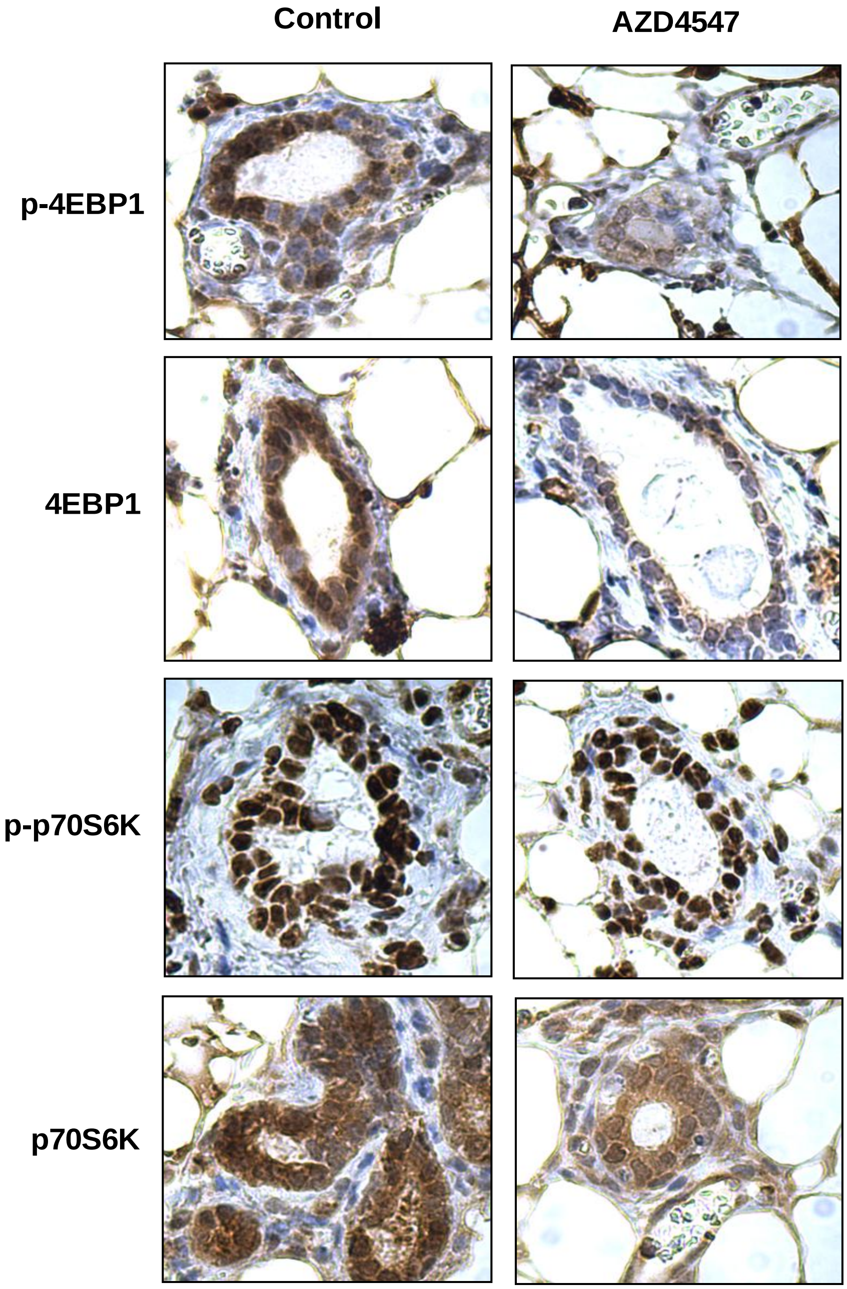

Supplement: Supplementary file 1 — Supplementary Information [file 41598_2017_11751_MOESM1_ESM.pdf]
